# Predicting anorexia nervosa treatment efficacy: an explainable machine learning approach

**DOI:** 10.1186/s40337-025-01265-3

**Published:** 2025-06-02

**Authors:** Giulia Brizzi, Chiara Pupillo, Elena Sajno, Margherita Boltri, Federico Brusa, Federica Scarpina, Leonardo Mendolicchio, Giuseppe Riva

**Affiliations:** 1 Department of Psychology, Università Cattolica del Sacro Cuore, Largo Gemelli, 20121 Milan, Italy; 2 Humane Technology Laboratory, Università Cattolica del Sacro Cuore, Largo Gemelli, 20121 Milan, Italy; 3 Department of Computer Science, University of Pisa, Pisa, Italy; 4 Experimental Laboratory for Metabolic Neurosciences Research, I.R.C.C.S. Istituto Auxologico Italiano, 28824 Piancavallo, VCO Italy; 5 “Rita Levi Montalcini” Department of Neurosciences, University of Turin, Turin, Italy; 6 U.O. di Neurologia e Neuroriabilitazione, Ospedale San Giuseppe, I.R.C.C.S. Istituto Auxologico Italiano, Piancavallo, VCO Italy; 7 Applied Technology for Neuro‐Psychology Laboratory, IRCCS Istituto Auxologico Italiano, 20149 Milan, Italy

**Keywords:** Machine learning, Anorexia nervosa, Eating disorders, Body image, Social relationships

## Abstract

**Introduction:**

Anorexia nervosa (AN) is a psychopathology with an alarmingly high mortality rate. The growing number of individuals seeking help, coupled with the limited resources of clinics, highlights the critical need to identify factors that can predict treatment efficacy. Machine learning (ML) techniques hold great promise in this regard. This data-driven approach offers an unbiased means to uncover predictors of specific outcomes, advancing the understanding and management of this challenging condition.

**Objective:**

Six supervised ML algorithms (e.g., Decision Tree and Random Forest) were applied to develop a binary classification model predicting short-term weight recovery/stabilization in AN inpatients and identify the most critical factors influencing this outcome.

**Methods:**

Change in Body Mass Index (BMI) from admission to discharge (ΔBMI) was used as the outcome, allowing to classify patients into “improved” (BMI stability or increase) and “aggravation” (BMI decrease). Predictors included clinically relevant psychological tests and physical parameters. Scikit-learn features importance, and SHAP (SHapley Additive exPlanations) analyses were used to investigate predictor importance.

**Results:**

The Random Forest model achieved an accuracy of 0.77, an AUC-ROC of 0.72, and a PR curve score of 0.88. Body Uneasiness, Personal Alienation, and Interpersonal Problems subscales emerged as best predictors. SHAP analysis confirmed these results at the individual prediction level.

**Discussion:**

Results encouraged interventions focused on body-self experience in addition to interpersonal relationships, including body-swapping experiences and metaverse activities, respectively. This could maximize treatment efficacy, effectively allocating limited resources to achieve clinically relevant outcomes.

**Supplementary Information:**

The online version contains supplementary material available at 10.1186/s40337-025-01265-3.

## Plain Language Summary

Anorexia nervosa (AN) is a serious eating disorder with one of the highest mortality rates of any mental illness. As the number of people seeking help increases, clinics face challenges in providing effective treatment due to limited resources. This study explored the use of supervised machine learning (ML) to predict weight change in a sample of inpatients with AN. Six supervised ML models were trained to predict changes in body mass index (BMI) from admission to discharge, categorising patients into two groups: "improving" (BMI stable or increasing at the end of hospitalisation) and "worsening" (BMI decreasing at the end of hospitalisation). After finding the best performing model, predictor importance extraction was performed to identify which psychological and physical parameters influenced BMI change. The Random Forest model was found to be the best model, with an accuracy of 77%. It highlighted specific psychological factors - such as body image dissatisfaction, feelings of personal disconnection, and relationship difficulties - as the most influential predictors of treatment outcome. These findings suggest that focusing on patients' body image and interpersonal relationships during therapy, including innovative approaches such as virtual reality and metaverse-based activities, may improve treatment success. This research highlights the potential of ML to tailor interventions for people with AN, ensuring that limited resources are used effectively to achieve the best possible outcomes.

**Supplementary Information:**

The online version contains supplementary material available at 10.1186/s40337-025-01265-3.

## Introduction

Anorexia Nervosa (AN) is a severe Eating Disorder (ED) marked by extreme self-imposed food restriction, significantly low body weight, intense fear of gaining weight, and a distorted body image. This distortion reflects an altered perception of the body and a dysfunctional relationship between body and self, where physical appearance heavily influences self-worth and identity [1, 2]. The development of AN arises from a complex interplay of biological, psychological, and sociocultural factors. Genetic variations influence metabolic and appetite-regulating hormones like leptin and dopamine [3]. Dysfunctional thought patterns about body weight, perfectionism, and novelty-seeking personality traits further increase vulnerability [4, 5]. Sociocultural pressures, including thinness ideals and parental attitudes toward food, also play a significant role [6, 7].

The interplay of these factors contributes to AN’s chronicity and severity, with studies showing that 19–26% of individuals meet diagnostic criteria even after 20–30 years [8]. This chronic nature makes AN a life-threatening mental health condition with severe physical and psychological implications. Recent research showed that AN affects up to 3% of young women and has the highest mortality rate of any psychiatric disorder, with approximately 5% of patients dying within four years of diagnosis [9, 10]. These epidemiological statistics portray AN as a public health emergency, particularly given that lifetime prevalence ranges from 0.5 to 3.5% in women and 0.1–2.0% in men [11]. The situation has worsened following the COVID-19 pandemic [12], which resulted in a 48% increase in hospital admission rates to specialized ED units compared to previous years [12].

In response to these concerning trends and the multifaceted nature of AN, the current situation presents significant challenges for healthcare systems worldwide. AN remains a challenging disorder to comprehend and treat, given its complex etiology and diverse manifestations, and the rising demand for healthcare providers and therapeutic interventions has placed strain on existing services. This pressing situation has steered the ED research field, particularly in anorexia, towards more efficient identification of outcome predictors and treatment efficacy factors. The goal is to strategically allocate limited resources to maximize clinical impact and help the greatest number of individuals effectively [13, 14].

Longitudinal studies investigated predictors of treatment outcomes in AN. For instance, Fichter et al. [15] identified Body Mass Index (BMI) at admission and Eating Disorder Inventory Maturity Fears subscale at admission as critical factors in determining the successful recovery—intended as the absence of AN diagnosis—in 112 patients. However, these types of studies are difficult to conduct due to high costs, low power, high attrition rates, and testing of specific or limited hypotheses (e.g. a limited set of predictors), leading to difficulties in replication and limited study comparability [10].

In recent years, technological advances have allowed for more sophisticated and effective techniques than clinical longitudinal research, thanks to Machine Learning (ML). ML facilitates the development of predictive models that can be used to test hypotheses, make inferences from data, and employ flexible, data-driven approaches to maximize predictive power [16, 17]. This study specifically focuses on supervised ML methods, where models are trained on labeled datasets containing input–output pairs with known outcomes. This approach involves selecting predictors based on existing literature and clinical expertise, defining an outcome label such as treatment effectiveness, and developing a model able to predict this label based on selected features [16]. In other words, supervised ML models detect patterns and relationships that traditional analysis might not detect. Supervised ML has been applied in emerging use cases in EDs domain, including risk factor identification, monitoring of patients, and predicting treatment response and prognosis in clinical populations (for a review, see [18]).

In the mental health research field, one line of research is applying ML models to predict treatment outcomes and identify critical factors linked to clinically positive outcomes [19]. For example, ML has been used to predict treatment outcomes for depressive disorder (for a review, see [20]). Chekroud et al. [21] employed supervised ML to predict patients' responses to antidepressant medication by analyzing clinical variables, including baseline symptom severity, treatment history, and sociodemographic information. In the field of cognitive rehabilitation for Mild Cognitive Impairment and dementia, ML techniques have been applied to predict disease progression, conversion from MCI to dementia, and rehabilitation outcomes starting from neuropsychological tests and sociodemographic data [22].

In the EDs research field, current research has primarily concentrated on two areas: (i) identifying biomarkers through neuroimaging (e.g., [23]) and (ii) detecting at-risk individuals (e.g., [24]). For instance, [25] reviewed the use of ML and Natural Language Processing methods to predict AN symptom from social media posts and comments, reporting relatively good performance levels (F1-score ranging from 0.67 to 0.93). Frank et al. [10] developed a ML model to predict BMI at a 6-month follow-up in a sample of individuals with AN treated in a 6-day per week partial hospital program, finding that BMI change from admission to discharge was the most important predictor, strongly correlating with BMI at follow-up (r = 0.55), while clinical questionnaire scores (e.g., State-Trait Anxiety Inventory, Beck Depression Inventory-II) were less predictive. Sandoval-Araujo et al. [26] applied ML to distinguish between typical and atypical AN using BMI, reinforcing its role as a key diagnostic parameter. Another line of research instead used ML methods to classify individuals with a history of AN at two stages of recovery from healthy controls using brain magnetic resonance images, observing differences in cortical thickness and gray matter volume across several regions (i.e., insula, lateral orbitofrontal, and temporal pole,[24]). Similarly, Lavignino et al. [23] identified six brain regions (i.e., cerebellum white matter, choroid plexus, putamen, nucleus accumbens, the diencephalon, and the third ventricle) to be relevant in distinguishing individuals with AN from healthy controls.

While ML applications in EDs, particularly AN, are emerging, their integration into clinical practice remains limited. Moreover, a major gap persists in studies investigating the key variables that influence treatment outcomes and long-term recovery trajectories. This underscores the need for broader ML applications to support clinical decision-making, treatment development, and personalized intervention strategies in AN care.

The present study aims to address this gap by expanding ML applications focusing on understanding factors that influence treatment outcomes in AN. Specifically, this research employs supervised ML techniques with two primary objectives:

- Create a model to predict the effectiveness of ED treatment in a cohort of hospitalized patients affected by AN,
- Identify the most influential physical and psychological variables for predicting positive treatment outcomes, specifically in terms of weight recovery.

A deeper understanding of the critical factors contributing to effective intervention programs can help target the core aspects influencing treatment outcomes. This allows for a more strategic allocation of limited resources (e.g., financial, personnel, and physical space) to enhance overall treatment outcomes and reach a broader population. Moreover, this approach offers valuable insights into AN mechanisms, stressing key variables that play a pivotal role through a data-driven perspective.

## Methods

### Data source

Data for this study was retrieved from the study by Brusa et al. [27]. It contains clinically relevant physical and psychological parameters assessed before starting the rehabilitation program at the IRCCS Istituto Auxologico Piancavallo (Italy).

The dataset contains information related to severe 72 patients (68 females, 4 males) with a diagnosis of AN (54 restrictive, 18 binge-purge subtypes) admitted to a multidisciplinary hospitalization program for EDs between August 2021 and July 2022. Inclusion criteria were: (a) a primary diagnosis of AN, (b) a BMI at admission equal or lower to 17 kg/m^2^, indicating moderate to extreme severity, and (c) compliance with the rehabilitation program. Table 1 presents sample descriptives.

**Table 1:** Sample descriptives

	Mean (sd)	Minimum	Maximum
Age	24.11 (12.41)	13	66
Duration of Illness (years)	10.14 (12.53)	0	58
Age first diagnosis (years)	16.23 (6.41)	10	50
BMI (admission)	14.13 (1.58)	9.73	16.9
BMI (discharge)	14.49 (1.45)	10.22	17.6
Hospitalization length (days)	35.83 (9.07)	21	59

BMI = Body Mass Index

Patients were exposed to a multidisciplinary treatment, proposing both individual and group activities (for a more detailed explanation of the treatment see Supplementary Materials—Table 8 -and the original study by [27]).

Clinically relevant parameters were assessed at the beginning of hospitalization (T0) and the end of the rehabilitation program (T1).

Because of the severity of patients (BMI mean = 14.13, sd = 1.58) and the short treatment length (Days mean = 35.83, sd = 9.07) we preliminary performed a paired sample t-test to investigate significant differences in BMI before and after the treatment. The analysis showed a significant difference, with the BMI ad admission being significantly higher than the BMI at the discharge (Table 2).

**Table 2:** Paired sample t—test

		Student’s t	df	*p*	Mean Diff (SE)	Cohen’s d
BMI (admission)	BMI (discharge)	− 3.92	71.0	< 0.01	− 0.35 (0.09)	− 0.46

The table presents the paired sample t test results to investigate differences between BMI at admission and discharge. SE = standard error, Diff = difference, df = degree of freedom

### Procedure

Procedure and results reporting were conducted based on Flanagin et al. [28] and Serino et al. [108].

#### Study design and outcome measure

We developed and evaluated ML models to predict treatment success for AN through binary classification of ΔBMI (i.e., weight improvement). Models were developed and trained to predict two different classes reflecting patients’ changes in BMI from admission to discharge (ΔBMI), where class 1 indicated weight recovery or stability (ΔBMI ≥ 0; 53 subjects) and class 0 indicated weight loss (ΔBMI < 0; 19 subjects). The choice of using BMI as an index of treatment effectiveness based on prior research [26, 30, 31]. Notably, our decision to consider BMI maintenance (no change) as a positive outcome is supported by research showing that severe anorexia (BMI < 15) and the restrictive subtype may have no change pre-post treatment in the short term [32]. Indeed, In severe cases of AN, maintaining weight can be a critical first step toward recovery: studies indicate that patients with extreme weight loss often experience significant physiological and psychological challenges that make immediate weight gain difficult. Then, stabilizing weight can prevent further health deterioration and provide a foundation for gradual recovery [33].

#### Features

The database contains clinical physical and psychological parameters commonly assessed in clinical practice. Below are presented main measures, whereas detailed information is provided in Supplementary Materials (Tables 5, 6).

##### Psychological variables

- The *Body Uneasiness Test* (BUT; [34]) is a 71-item questionnaire to assess body-related discomfort. It comprises two main subscales: subscale A refers to weight phobia, body image concerns, avoidance, compulsive self-monitoring, detachment, and depersonalization from the body (34 items), whereas subscale B measures worry related to different body parts (37 body areas). Examples of items of scale A include: “I spend a lot of time in front of the mirror” and “I feel I am fatter than others tell me”, whereas scale B asks participants to rate how much they hate different body areas such as height, arms, and stomach. This questionnaire has been validated in the Italian language, with a Cronbach’s alpha between 0.69 and 0.90 [34].
- The *Eating Disorder Inventory-3* (EDI-3; [35]) is a 91-item questionnaire that assesses three ED-specific pathological symptom categories (drive for thinness, bulimia, body dissatisfaction) and nine general symptom categories (low self-esteem, personal alienation, interpersonal insecurity, interpersonal alienation, interoceptive deficits, emotional dysregulation, perfectionism, asceticism, maturity fears),additionally, 6 composite scales can also be calculated to characterize better the condition (eating concerns composite, ineffectiveness composite, interpersonal problems composite, affective problems composite, overcontrol composite, global psychological maladjustment). Examples of items include “I eat sweets and carbohydrates without feeling nervous” and “I think my stomach is too big”. The EDI-III has been validated in Italian language with Cronbach's alpha values for the subscales in ranging from 0.70 to 0.94.
- The *Psychological General Well-Being Index questionnaire* (PGWBI; [105]) is a 22-item questionnaire to assess Quality of Life (HRQoL). It comprehends six subscales: anxiety, depression, positivity and well-being, self-control, general health, and vitality. Examples of items are “How often were you bothered by any illness‚ bodily disorder‚ aches or pains during the past month?” and “Did you feel depressed during the past month?”. It has been validated in the Italian language with Cronbach's alpha coefficients ranging from 0.92 to 0.94.
- The *Symptom Checklist-90* (SCL-90; [36]) is a 90-item questionnaire to assess nine symptomatologic dimensions: somatization; obsessive–compulsive behaviours and thoughts, interpersonal sensitivity, depression, anxiety, hostility, phobic anxiety, paranoid ideation, and psychoticism. Items are rated on a 5-point Likert scale from 0 (not at all) to 4 (extremely). Examples of items include the rating of how much participants were bothered by “headaches” and “feeling others are to blame for most of your troubles”. The questionnaire has been validated in the Italian language, showing Cronbach's alpha coefficients between 0.70 and 0.96.
- *The Frost Multidimensional Perfectionism Scale* (FMPS; [37]) is a 35-item questionnaire assessing five dimensions of perfectionism, namely personal standards, concern over mistakes, parental expectations, doubting of actions, and organization. Examples of items include “I feel that I have made a lot of mistakes in my life" and "If I fail at work/school, I will be a failure as a person”. The questionnaire has been validated in the Italian language, showing Cronbach's alpha ranging from 0.86 to 0.89.

##### Physical variables

- The *Bioelectrical impedance analysis* (BIA; [107]) is a method used to estimate body composition focused on the amounts of body fat and muscle mass. This technique relies on measuring the body’s impedance, namely the body's opposition to the flow of an electric current (i.e., resistance and reactance). From this parameter, it is possible to calculate extracellular water, body cell mass, and phase angle.

The final features set consisted of 52 features, including questionnaire subscale scores and physical parameters assessing patients' state at the beginning and the end of the hospitalization. For this study, only parameters at the beginning of the treatment program were considered.

A more in-depth explanation of predictors (features) is available as Supplementary Material.

The model did not include demographic variables based on Frank et al. [10].

#### Data preparation and feature selection

The dataset was initially examined for quality and completeness. Missing values were imputed using the K-Nearest Neighbors Imputer, which has shown robustness in handling missing data in clinical datasets [38]. All features were scaled to the same values using StandardScaler, which is considered a necessary practice to improve the accuracy of ML model predictions [39]. The Variance Inflation Factor (VIF) was calculated for each predictor variable to assess multicollinearity. No variables exhibited a VIF greater than 10, indicating that multicollinearity was not a significant concern in our dataset [109].

#### Machine learning pipeline

**Fig. 1:**
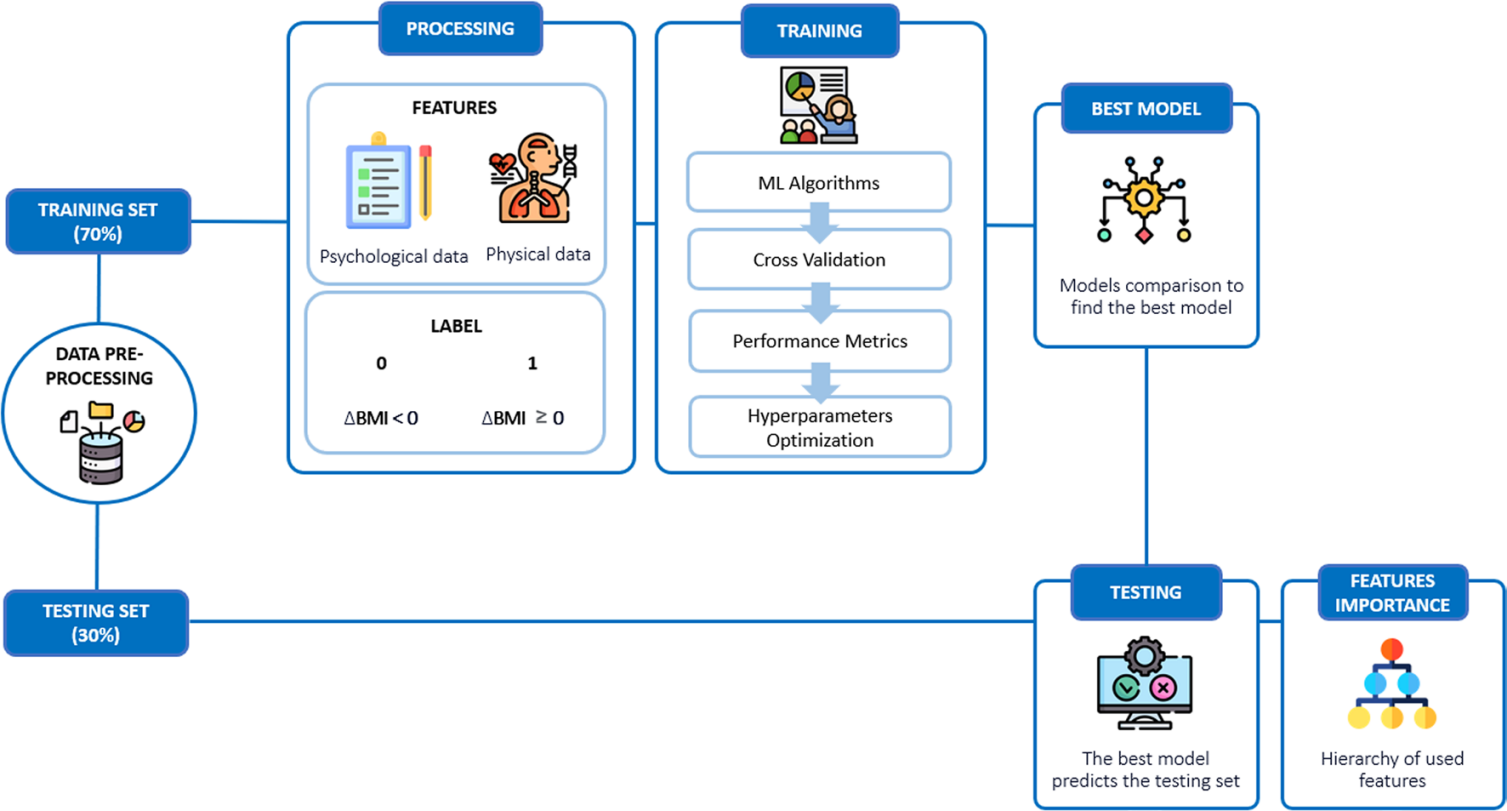
Machine Learning pipeline. The figure graphically summarizes the machine learning steps

ML pipeline (see Fig. 1) started with data preprocessing and cleaning with subsequent delta BMI label calculation. A Dummy Classifier (DC) was initially generated as a baseline model to establish a performance benchmark for randomness. In addition, Linear Discriminant Analysis (LDA) was applied to compare classification performance with the other models [40].

Subsequently, six supervised ML algorithms were trained based on ones commonly used in the EDs research field: specifically, Decision Tree (DT), Random Forest (RF), Gradient Boosting (GB), Support Vector Machine (SVM), Logistic Regression (LR), and k-Nearest Neighbors (kNN) were tested based on Gosh et al. [18].

The dataset was randomly split into a 70/30 ratio, with 70% allocated for training to allow the models to learn underlying patterns and 30% reserved for testing to evaluate their generalization ability on unseen data. This split follows standard ML practices, balancing the need for sufficient training data while ensuring a robust test set for performance assessment [41]. fivefold and tenfold cross-validation with 5 repeats were performed for each algorithm to measure the models’ performance. This process allowed the assessment of model stability and generalizability across different data subsets. Following the cross-validation phase, hyperparameter optimization for all six algorithms was performed by using three main techniques: Randomized Search, Bayesian Search, and Grid Search [42]. This phase aimed to refine the parameters of each model to achieve optimal performance. After this, a new cross-validation on the optimized models was performed to assess the performance improvement.

Based on optimization results and cross-validation processes, the two best-performing models were selected for final testing on the held-out test set. Importance features analyses were performed for the best model to identify the most important predictors. Finally, to enhance model interpretability, SHAP (SHapley Additive exPlanations) was utilized to understand the contribution of individual features to the model’s predictions.

#### Model evaluation

The evaluation metrics considered for each phase of the training and testing set were accuracy (i.e., the ratio between the correctly classified samples and the total number of samples), precision (i.e., the ratio between correctly classified samples and all samples assigned to that class), recall (i.e., sensitivity or True Positive Rate, indicating the ratio between correctly classified positive samples and all samples assigned to the positive class), specificity (i.e., the ratio between correctly classified negative samples and all samples classified as negative), F1 score (i.e., harmonic mean of precision and recall), and Area Under the Curve—Receiver Operating Characteristic (AUC-ROC—i.e., ROC curve summary metric that reflects the ability to distinguish between classes, shows the overall diagnostic accuracy). These metrics were selected as they are the most widely used in binary classification in clinical research [43], including in EDs [18, 26]. Confusion matrix was also generated to provide a detailed breakdown of the model predictions. Finally, the area under the curve precision-recall (PR-AUC) was assessed to address potential imbalances between classes and obtain an additional metric of overall performance [44].

#### Model validation

A repeated stratified K-fold cross-validation was employed to evaluate the model effectively. This splits the data set into multiple folds to train and test the model iteratively, providing a more reliable performance measure than a single training-test split. The K-fold variant splits the data into K subsets, trains the model on K-1 folds, and tests it on the remaining fold, repeating this process K times. Stratification ensures that the proportion of positive and negative samples is consistent across folds, which is critical for unbalanced data sets because it preserves class distributions. It has been seen to be one of the most suitable cross-validation types for small clinical datasets with multi-characteristic subjects, ensuring a balanced trade-off between bias (errors from overly simplistic models that do not fit the data) and variance (errors from excessively complex models that overfit the data) [45, 46]. In addition, it has been seen that smaller K values (10, 5, 3) for small sizes offer a practical compromise, balancing computational efficiency and reliability of performance estimates [45].

#### Feature importance analysis

Feature importance analysis was performed using the feature_importances_ attribute from scikit-learn to specifically evaluate individual predictors’ impact on the best performing model and extract the 10 most influential features. These importance scores were computed as each tree’s mean and standard deviation of the impurity decreased. Specifically, higher relative importance scores reflected features that are most relied on by the model to make predictions.

In addition, the SHAP (SHapley Additive exPlanations; [47]), Explainable AI (XAI) method was used to make the results more interpretable. SHAP was used because it assigns an importance value to each feature in the model output and is designed to be applied a posteriori to any type of ML model (Lundberg and Lee, [48]),the 20 most relevant parameters are extracted by default (Lundberg & Lee, [48]). In this context, Tree. Explainer algorithm was used since it is suitable for tree models such as Random Forest. SHAP values also provide a deeper understanding of the contributions of characteristics to individual predictions. SHAP summary plots were created for the overall model and separately for the two AN subtypes (0 = restrictive, 1 = binge-purge). This subtype-specific analysis was conducted solely as an additional post-hoc interpretability using SHAP values. No additional ML training was performed for the subtypes because the limited sample size precluded the development of separate ML models for each subtype.

Each patient was represented by a single point for each feature in the graph. The x-axis coordinate of each point was determined by the SHAP value, and the points were stacked along each feature to show their density. The features were sorted by the average absolute value of the SHAP values for each feature. Color was used to indicate the original value of a feature; red and blue indicate the high or low of the individual feature, respectively. The gray vertical line of the decision graph represents the baseline value of the model.

To compare and integrate the results of these two methods, it is essential to note that SHAP values provide a measure of how much each feature contributes to the model's output for a particular prediction, indicating both positive and negative impacts. In contrast, feature importance rankings generally focus on the overall importance of each feature in the model, providing only those that impacted positively. Consequently, discrepancies in the order of importance between the two methods may arise.

ML algorithms training and testing were carried out in Python v. 3.10.12, using Google Colab v. 0.0.1a2. We used the following Python packages: *numpy, pandas, matplotlib, scikit-learn, seaborn, scipy.stats, scikit-optimize*, and *shap*.

## Results

### ML performance evaluation during training and testing to predict delta BMI

The six trained ML algorithms ten folds cross-validation performance is reported in Table 3. Notably, all models outperformed our benchmark for randomness, namely DC model (AUC-ROC = 0.50) and LDA (accuracy = 0.31; [49]). DC and LDA classification reports are reported as Supplementary Material.

**Table 3:** Evaluation metrics of the tenfold with 5 repeats cross-validation for the six algorithms

	Decision Tree	Random Forest	Gradient Boosting	Support Vector Machine	Logistic Regression	k-Nearest Neighbors
Accuracy	0.70 (0.18)	0.70 (0.15)	0.71 (0.18)	0.74 (0.09)	0.58 (0.22)	0.67 (0.19)
AUC-ROC	0.61 (0.23)	0.65 (0.27)	0.65 (0.27)	0.62 (0.30)	0.57 (0.31)	0.65 (0.26)
Precision	0.80 (0.19)	0.75 (0.14)	0.81 (0.18)	0.74 (0.09)	0.71 (0.21)	0.74 (0.16)
Recall	0.79 (0.23)	0.91 (0.15)	0.82 (0.20)	1.00 (0.00)	0.71 (0.26)	0.84 (0.20)
Specificity	0.43 (0.45)	0.14 (0.33)	0.44 (0.47)	0.00 (0.00)	0.24 (0.40)	0.18 (0.36)
F1-Score	0.77 (0.18)	0.81 (0.12)	0.79 (0.15)	0.85 (0.06)	0.69 (0.20)	0.78 (0.16)

The values are expressed as mean values and their standard deviation in brackets. AUC-ROC = Area Under the Curve—Receiver Operating Characteristic

The best hyperparameters were found with Bayesian Optimization, which obtained similar values to the other two methods and is the most robust technique. As shown in Table 4, the best-performing algorithms trained with the fitted hyperparameters were Decision Tree (DT) and Random Forewst (RF).

**Table 4:** Evaluation metrics of the tenfold with 5 repeats cross-validation for the two optimized models: Decision Tree and Random Forest

	Decision Tree	Random Forest
Accuracy	0.72 (0.20)	0.76 (0.15)
AUC-ROC	0.68 (0.23)	0.67 (0.23)
Precision	0.85 (0.17)	0.88 (0.12)
Recall	0.75 (0.26)	0.81 (0.23)
Specificity	0.60 (0.44)	0.60 (0.44)
F1-Score	0.77 (0.21)	0.81 (0.14)

The values are expressed as mean values and their standard deviation in brackets. AUC-ROC = Area Under the Curve—Receiver Operating Characteristic

The testing phase was conducted exclusively on the two best-performing models, DT and RF, which were identified based on their superior performance during training. To assess their generalization ability, we evaluated these models on a previously unseen 30% hold-out test set from the original dataset. This test set was not used during training, ensuring that the evaluation reflects how well the models can classify new data in our binary classification. The results of the final best model—RF—are presented in Table 5: its results show an accuracy of 0.77, highlighting the model’s ability to correctly classify most of the cases in the test set.

**Table 5:** Evaluation metrics of the testing set for the optimized Random Forest (RF) model

Random forest—test		
	Class 0 (negative Δ BMI)	Class 1 (0 or positive Δ BMI)
Accuracy	0.77	
AUC-ROC	0.72	
Precision	0.67	0.79
Recall	0.33	0.94
Specificity	0.33	0.33
F1-Score	0.76	0.86

The table shows the results for both binary classes. AUC-ROC = Area Under the Curve—Receiver Operating Characteristic

**Fig. 2:**
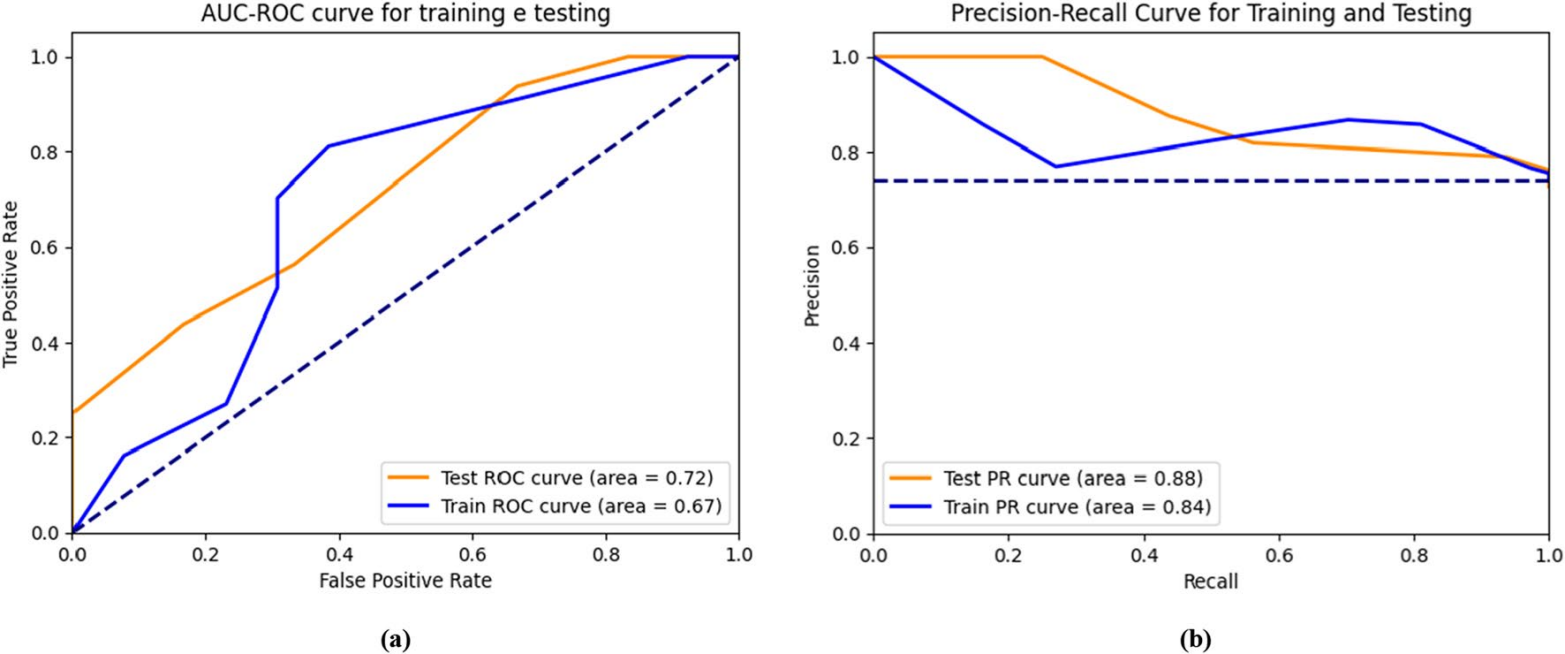
Visual comparison of a performance model for training and testing set for Random Forest. The blue lines represent the training curves, the orange lines represent the testing curve, and the dashed lines of a random classifier. A higher curve represents a better performance of the model, to be acceptable it must exceed the dotter lines. The first figure represents the AUC-ROC curve (**a**). The AUC-ROC curve shows the relationship between the true positive rate and the false positive rate. The second figure represents the Precision-Recall (PR) curve (**b**). The PR curve shows the relationship between precision and recall. AUC-ROC = Area Under the Curve—Receiver Operating Characteristic

To evaluate the RF performance, its ability to predict class 1 and 0 were tested. The AUC-ROC of 0.72 indicated a fair discrimination without overfitting and outperforming the training curve (AUC-ROC = 0.67; Fig. 2a). The curves' shapes indicate that the model performs consistently better than random chance across various classification thresholds. Figure 2b presents the Precision-Recall (PR) curves for training and testing. The PR-AUC values for testing (0.88) and training (0.84) are notably high, indicating robust performance in balancing precision and recall.

**Fig. 3:**
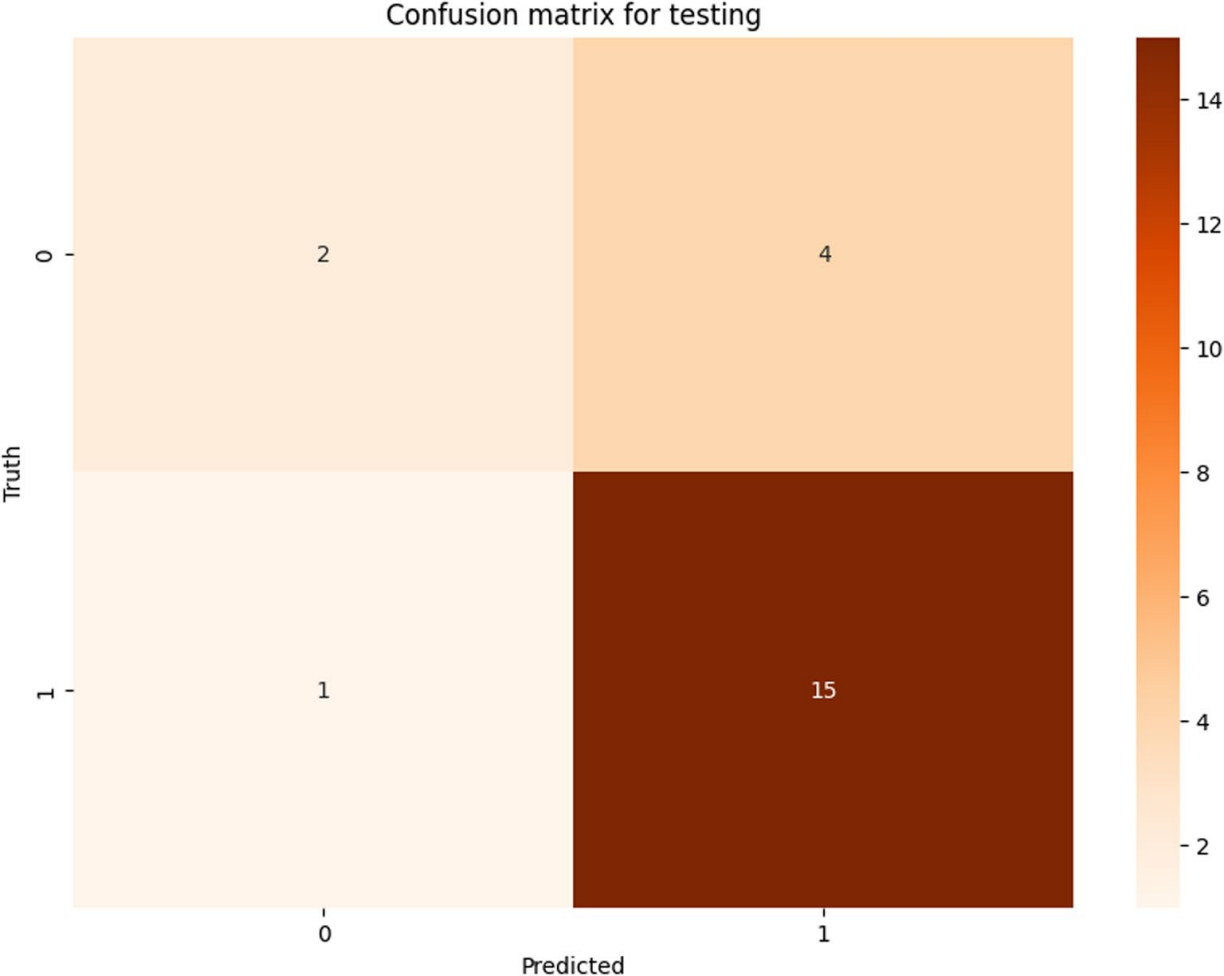
Confusion matrix of testing set. The picture shows the four basic characteristics for evaluating a classification model. In this case it concerns the confusion matrix of the best model (Random Forest) for testing set. 1 indicated weight recovery or stability (ΔBMI ≥ 0) and 0 indicated weight loss (ΔBMI < 0). In the first row, the first cell indicates the true negatives (number of patients correctly classified that their delta BMI has worsened) and the adjacent cell the false positives (number of patients misclassified with improved or unchanged delta BMI, known as type I error), while the second row shows the false negatives (number of patients who were misclassified with improved or unchanged delta BMI but actually had negative delta BMI, known as type II error) and true positives (number of patients who were correctly classified as patients to whom delta BMI was positive or unchanged), respectively

The model correctly identified expected positives (precision = 0.79) and true positives (recall = 0.94). However, it showed difficulties in identifying true negatives (specificity = 0.33). Similarly, the confusion matrix (Fig. 3) showed that the model accurately classified 15 out of 16 positive cases (True Positives), but struggled with identifying negative outcomes (Class 0), misclassifying 4 out of 6 as positives (False Positives). Despite the 4 false positives, the high precision (0.79) is explained by the large number of true positives (15) relative to false positives (4). The high recall and precision for Class 1 suggest that the model is highly reliable in predicting positive BMI changes, which are critical for assessing treatment success. On the other hand, the high false positive rate and low specificity is given by the imbalance of the two classes, as the negative class is lower than the positive class [50].

### Predictors importance

**Fig. 4:**
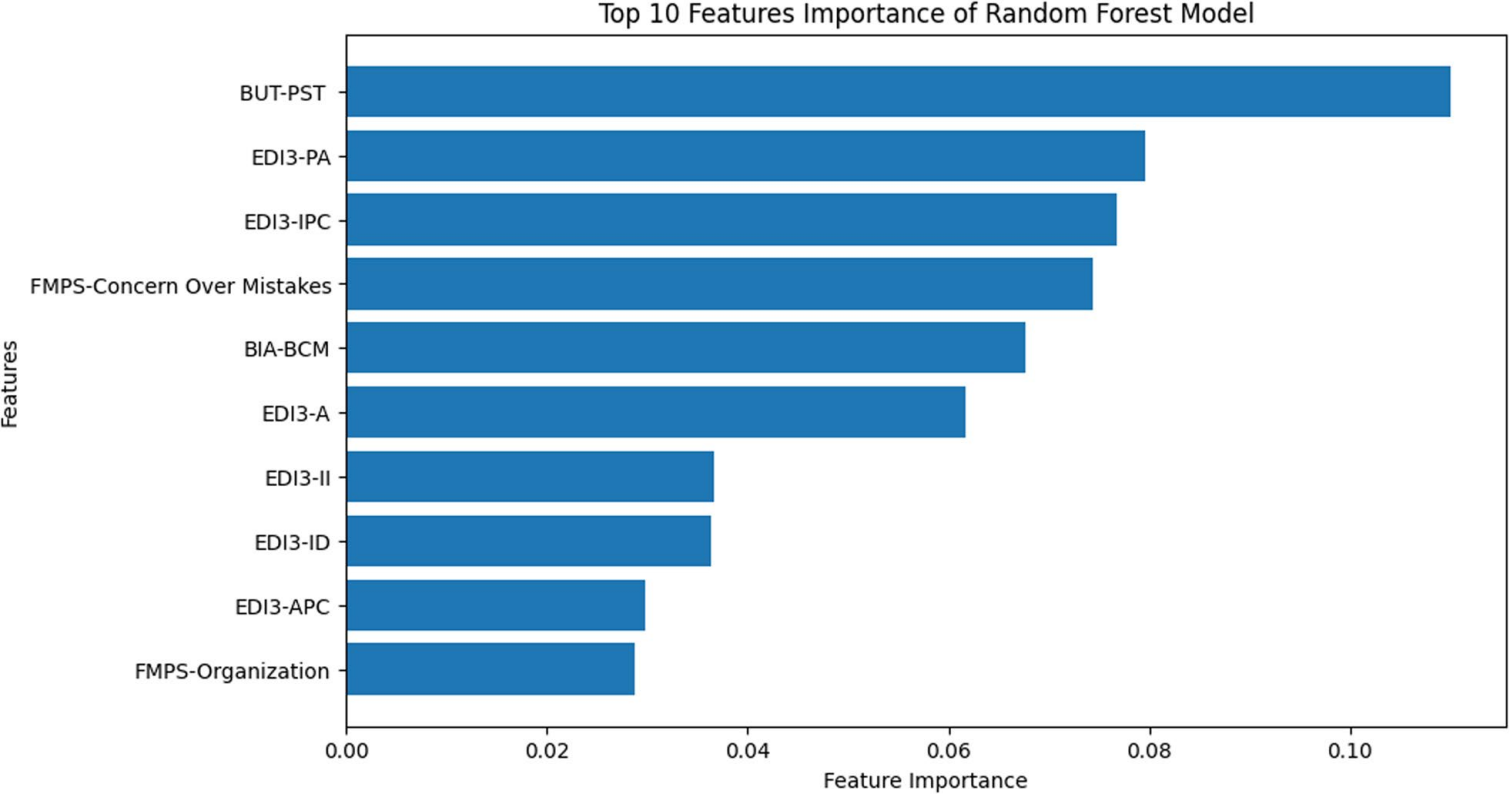
Scikit-learn-based feature importance rank for predicting class 1 (0 or positive delta BMI). The features importance plot shows the 10 most important predictors of weight recovery and/or stability. BUT = Body Uneasiness Test, EDI3 = Eating Disorder Inventory 3, FMPS = Frost Multidimensional Perfectionism Scale, BIA = Bioelectrical impedance analysis. BUT-PST = Positive Symptom Total, EDI3-PA = Personal Alienation, EDI3-IPC = Interpersonal Problems Composite, BIA-BCM = Body Cell Mass, EDI3-A = Asceticism, EDI3-II = Interpersonal Insecurity, EDI3-ID = Interoceptive Deficits, EDI3-APC = Affective Problem Composite

Initially, the importance of the features of the optimized RF model was evaluated using feature importance analysis, which showed the 10 most important model predictors for the training set. BUT-PST, EDI3-PA, and EDI3-IPC resulted as the top three most influential parameters (Fig. 4).

**Fig. 5:**
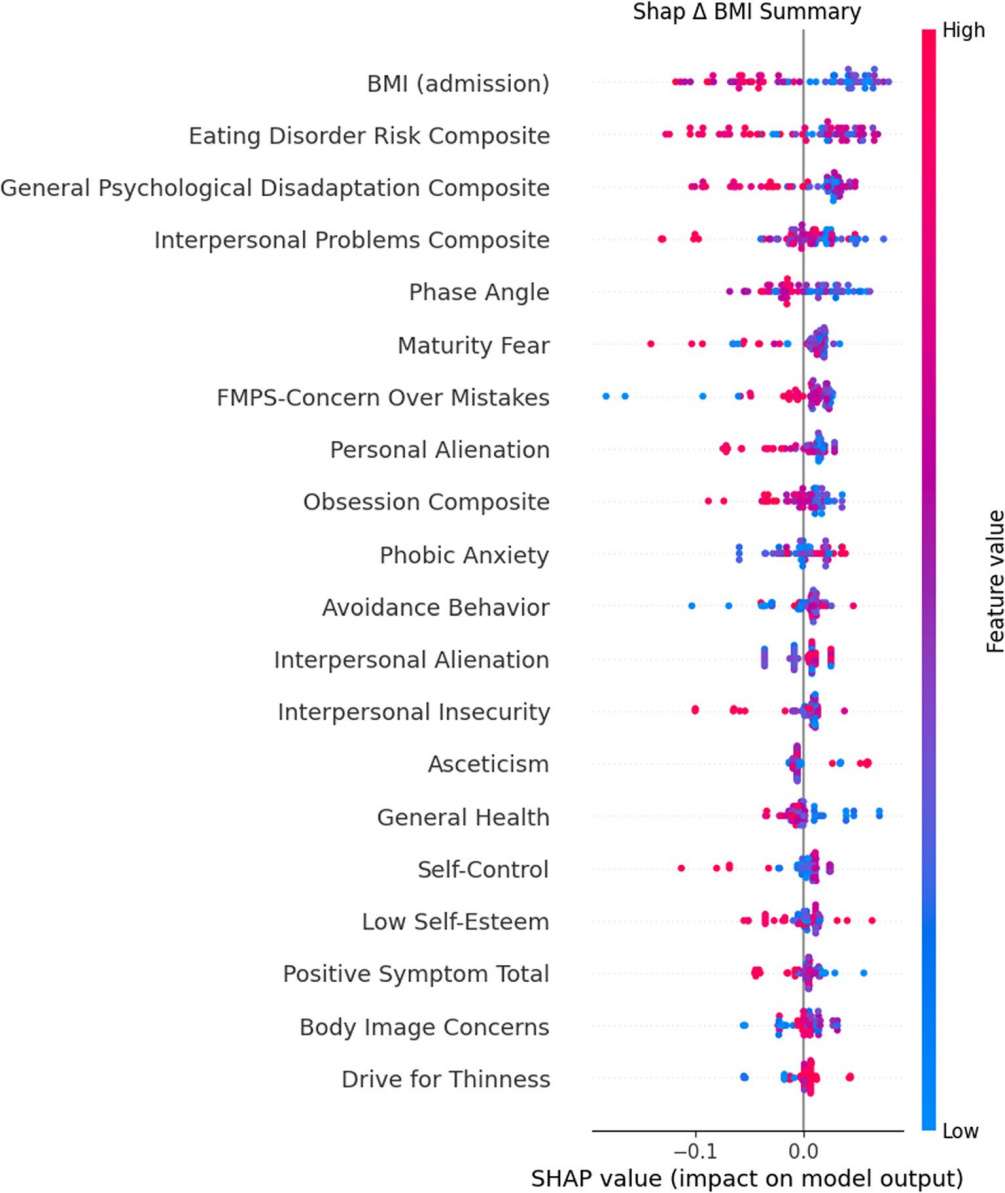
Summary plot of SHAP-calculation for the 20 highest-ranking features. Features are sorted by their mean absolute SHAP value in descending order with the most important variables at the top. Each dot corresponds to one patient in this study. This plot shows how the different variables of each patient affect the prediction of the RF model towards class 1 (0 or positive delta BMI). Positive SHAP values (to the right) indicate features that increase the likelihood of a positive BMI change, while negative SHAP values (to the left) indicate features that decrease this likelihood. Instead, the gradient of colors indicates positive (red) or negative (blue) values compared to the original feature values for each patient. The plot is based on the RF model with all features included for all 72 patients. BUT = Body Uneasiness Test, EDI3 = Eating Disorder Inventory 3, FMPS = Frost Multidimensional Perfectionism Scale, BIA = Bioelectrical impedance analysis, SCL90 = Symptom Checklist-90, PGWBI = Psychological General Well-Being Index questionnaire, BMI = Body Mass Index, EDI3-EDRC = Eating Disorder Risk Composite, EDI3-GPMC = General Psychological Disadaptation Composite, EDI3-IPC = Interpersonal Problems Composite, BIA-PA = Phase Angle, EDI3-MF = Maturity Fear, EDI3-PA = Personal Alienation, EDI3-OC = Obsession Composite, BUT-A = Avoidance Behavior, EDI3-IA = Interpersonal Alienation, EDI3-II = Interpersonal Insecurity, EDI3-A = Asceticism, EDI3-LSE = Low Self-Esteem, BUT-PST = Positive Symptom Total, BUT-BIC = Body Image Concerns, EDI3-DT = Drive for Thinness

To further understand the impact of features on individual predictions, the SHAP technique was applied. The overall SHAP summary plot (Fig. 5) highlighted BMI (admission) as the most impactful feature, in addition to EDI3-EDRC and EDI3-GPMC.

Comparing the Scikit-learn-based feature importance rank and the SHAP outputs, EDI3-PA, EDI3-IPC, FMPS-Concern Over Mistakes remained in the top 10 predictors, while BUT-PST and EDI3-A were still present but in lower positions.

**Fig. 6:**
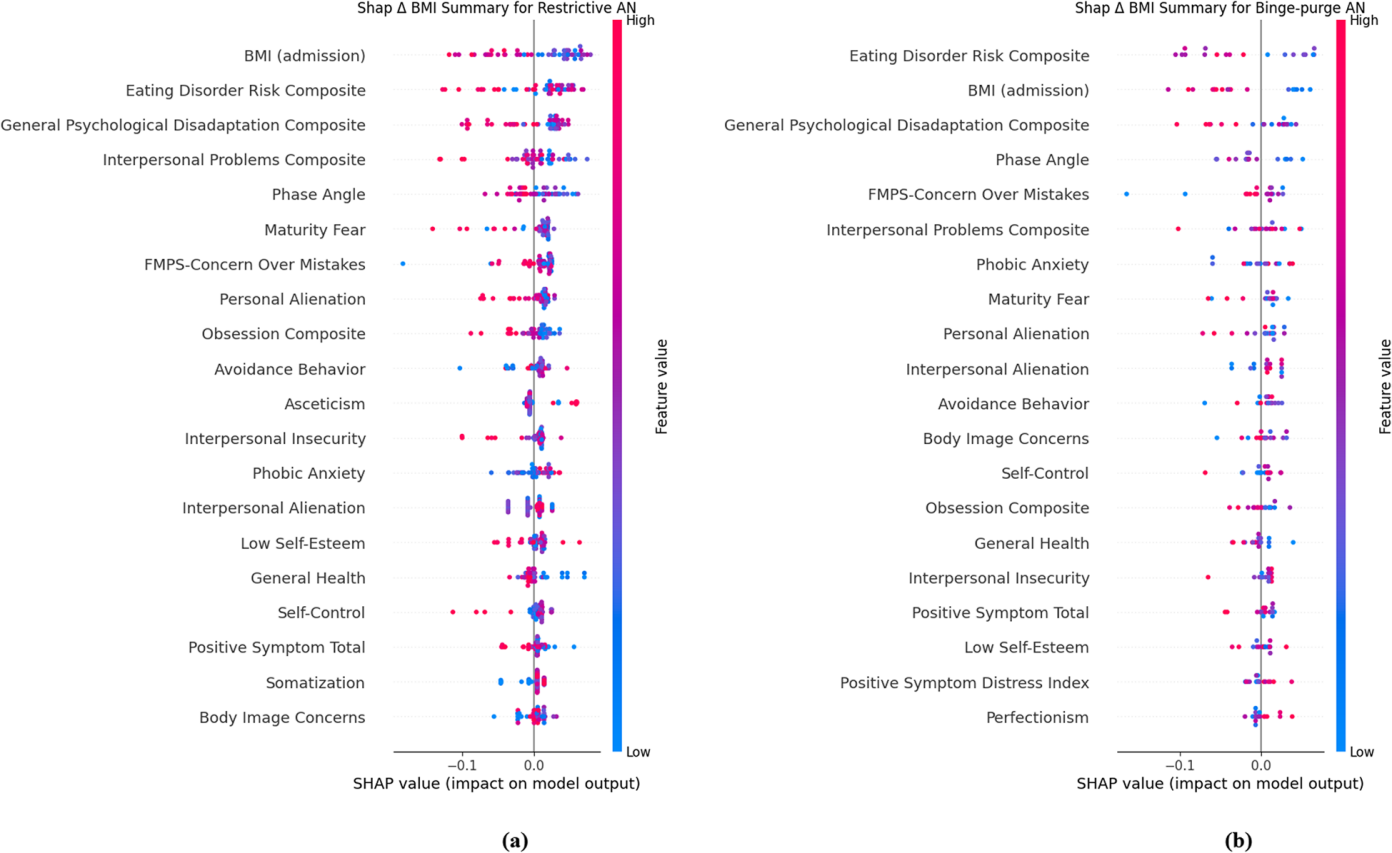
**a**, **b** Summary plot of SHAP-calculation for the 20 highest ranking features for subtypes AN. Plot shown for restrictive AN (**a**) and binge-punge AN (**b**). Features are sorted by their mean absolute SHAP value in descending order with the most important variables at the top. Each dot corresponds to one patient in this study. Positive SHAP values (to the right) indicate features that increase the likelihood of a positive BMI change, while negative SHAP values (to the left) indicate features that decrease this likelihood. Instead, the gradient of colors indicates positive (red) or negative (blue) values compared to the original feature values for each patient. The plot is based on the RF model with all features included for all 72 patients. Body Uneasiness Test = BUT, Eating Disorder Inventory 3 = EDI3, Frost Multidimensional Perfectionism Scale = FMPS, Bioelectrical impedance analysis = BIA, Symptom Checklist-90 = SCL90, Psychological General Well-Being Index questionnaire = PGWBI, Body Mass Index = BMI, Eating Disorder Risk Composite = EDI3-EDRC, General Psychological Disadaptation Composite = EDI3-GPMC, Interpersonal Problems Composite = EDI3-IPC, Phase Angle = BIA-PA, Maturity Fear = EDI3-MF, Personal Alienation = EDI3-PA, Obsession Composite = EDI3-OC, Avoidance Behavior = BUT-A, Asceticism = EDI3-A, Interpersonal Insecurity = EDI3-II, Interpersonal Alienation = EDI3-IA, Low Self-Esteem = EDI3-LSE, Positive Symptom Total = BUT-PST, Body Image Concerns = BUT-BIC, Positive Symptom Distress Index = BUT-PSDI, Perfectionism = EDI3-P

A more detailed posteriori analysis was performed on the two subtypes of AN, i.e. restrictive (R) (Fig. 6a) and binge-purge (BP) (Fig. 6b). In both conditions, BMI at admission emerged as the most relevant predictor, as observed in the overall SHAP model. Specifically, low-to-medium initial BMI predicted an equal to zero or positive ΔBMI. No differences emerged when comparing the R and the overall SHAP model, whereas differences emerged when considering the BP model. Here, features showed different feature rankings despite variables being the same.

## Discussion

The primary aim of this study was to develop a ML model to predict weight recovery or stabilization in a cohort of AN inpatients. Results from this study showed ML good performance levels (accuracy = 0.77, AUC-ROC = 0.72, and PR curve = 0.88), similar to previous studies (AUC-ROC values between 0.49 and 0.93, and accuracy between 0.59 and 0.86; [18, 51], Haynos et al., [52]). Despite differences in outcome measures, predictor variables, and performance metrics, the results of this study remain within the performance range reported in the literature, highlighting the potential clinical utility of this model in the context of eating disorders.

The secondary aim of this study was to identify which variables better predicted weight recovery, as a proxy of successful treatment. Scikit-learn feature importance extraction [53] and SHAP values [54] were used to identify predictors at the global and individual levels respectively. Interestingly, results were consistent: overall predictors were also reflected at the individual level, thus reinforcing the robustness of results [55]. The subsequent section goes into more detail concerning the three most important weight stability and recovery predictors. To conclude, we critically discussed the implications of integrating ML into clinical practice.

This study represents a preliminary, proof-of-concept effort designed to highlight the potential of ML methods in the field of EDs research. The following sections will explore the model's most significant predictors to conclude with a critical evaluation of the advantages and challenges associated with applying ML in clinical settings.

### Predicting weight recovery or stability

#### Body-self relationship

Scikit-learn features importance and SHAP analyses identified BUT score as a significant predictor of weight stability and recovery. In particular, the Positive Symptom Total (BUT-PST), Body Image Concerns (BUT-BIC), the overall Positive Symptom Distress Index (BUT-PSDI), and body-related concerns and dissatisfaction with own body image (BUT-A) emerged as critical factors. This aligns with early Bruch’s [56] insights, according to which AN interventions should carefully consider how patients perceive and relate to their bodies to be effective in the long term.

A large amount of evidence showed that AN is fundamentally characterized by a distorted experience of one’s body. This Body Image Disturbance goes beyond mere visual (mis)perception, representing a profound disconnection between the actual physical state and the subjective experience of their body at perceptual, emotional and cognitive levels [57, 58]. Specifically, research has shown that patients with AN report high levels of body dissatisfaction and body-related distress, which are reinforced by the internalization of unhealthy beauty standards [58]. Concerns still remain regarding perceptual alterations: while some studies reported individuals with AN significantly overestimate their body dimensions compared to healthy controls [59], others have found no such perceptual distortion [60]. Such inconsistency may partly stem from methodological differences across studies—e.g., different assessment tools ranging from explicit questionnaires to implicit measures—as well as limited carefulness in capturing bodily experience complexity.

In fact, how we experience our body depends on both emotions and beliefs, but also on how we process and integrate information from inside (interoceptive) and outside our body (exteroceptive). Recent studies revealed deficits in patients with AN both in processing inner body information (Lucherini et al., [118]) and at the level of integration of exteroceptive and interoceptive information [4]. This favors the idea of perceptual alterations that may reinforce body image disturbance, creating a self-reinforcing cycle of distorted body experience.

Further investigation of these perceptual mechanisms could reveal novel patterns and inform therapeutic approaches. For instance, impaired interoceptive awareness might create a cascade effect where disconnection from internal bodily signals leads to distorted cognitive interpretations—such as feeling bloated despite no physiological basis—ultimately contributing to body dissatisfaction.

Despite these insights into the multifaceted nature of body experience, standard intervention protocols in hospital settings tend to neglect the role of the body in AN, prioritizing the work on food-related behaviors and weight recovery through dietary management [58]. While this remains essential, evidence seems to suggest this focus alone may be insufficient for comprehensive recovery. From this perspective, Then, it is not surprising that, to date, there are no superior treatment options for AN. Established interventions—including Cognitive Behavioral Therapy (CBT) and Family-based therapy—show similar outcomes, with limited success in achieving clinically significant improvements [61]. Some authors have suggested that this therapeutic ceiling may stem from insufficient attention to body experience in conventional treatments [4, 58]. In response, an emerging line of research is exploring the integration of body image-centered protocols into AN treatment. These innovative approaches encompass multiple strategies: psycho-educational sessions (e.g., reasoning about body-checking behaviors, or social media use; [62, 63]), meditative practices (e.g., yoga, meditation,[64, 65]) or self-monitoring journaling exercises following Cognitive Behavioral Therapy Enhanced approach (CBT-E,[66]). Initial findings suggest that addressing altered body-self relationships positively influences recovery outcomes. Notable initiatives such as the Body Project [67] and The Body Wise [119] have further advanced this approach by developing experimental body-focused protocols specifically for AN treatment.

Recently, technology-based treatments have been also proposed, including Mirror Exposure [106] and Body Swapping (Serino et al., [29]) in Immersive Virtual Reality (IVR) environments. Such approaches use multisensory technology to place patients in bodies different in shape and weight from real ones, with the primary goal of proposing behavioral experiments that would not be possible in physical reality. For instance, in IVR Mirror Exposure paradigms, patients embody a normal-weight body and look at themselves in a virtual mirror from a third-person perspective [106]. This experience was observed in the fear of gaining weight [106]. Similarly, during Body Swapping procedure patients are asked to embody a normal weight body (BMI = 18.5), but this time from a first-person perspective [110]. Interestingly, this experience has been observed to reduce body distortion, as the swap into a virtual body helped patients to re-establish a connection with their physical bodies (Serino et al., [29]). Recently, our research group proposed two different protocols in this direction: the study by Malighetti al et al. [68] used a combination of IVR body swapping and mirror exposure into different bodies while asking patients to recall positive, negative, and neutral autobiographical memories depending on the body size (overweight, underweight, and normal-weight respectively, whereas the study by Brizzi and colleagues [120] (pre-print asked patients to recall what each part of her bodies was able to do for them in terms of enjoying full activities (i.e., functional mirror exposure. Following this trend, Regenerative Virtual Therapy (RVT; Riva et al., [121] has been proposed. It is an innovative therapeutic approach that combines principles from neuroscience, psychology, and advanced technology to restructure faulty bodily self-representations. What makes this proposal particularly interesting is the combination of multisensory technologies to reshape both the inner and outer body self-perception, brain-stimulation techniques and mindfulness practices.

Drawing from Cognitive Behavioral Therapy (CBT) principles, Immersive Virtual Reality (IVR) approaches recognize the pivotal role of behavior in influencing emotional states and thought patterns. Just as psychological disorders often involve maladaptive behaviors that reinforce dysfunctional emotions and cognitions, IVR provides a unique platform for behavioral modification. By enabling controlled behavioral interventions in virtual environments, IVR facilitates bottom-up therapeutic change, where behavioral alterations can catalyze cognitive restructuring [69].

The findings from this study underscore the critical importance of integrating body-focused interventions into AN treatment protocols to optimize weight recovery and maintenance outcomes. Innovative technologies like IVR, when combined with traditional therapeutic approaches, offer unique opportunities to address the complex interplay between emotional, cognitive, and perceptual components of body experience.

#### Social and interpersonal factors

The feature importance analysis revealed two other two key predictors in addition to body-self relationship: the EDI3-PA (Personal Alienation) and EDI3-IPC (Interpersonal Problems). This aligns with previous studies showing that interpersonal difficulties and relationship style (e.g., Non-assertive and Friendly-submissive interpersonal style) influence AN symptomatology onset and maintenance [70–72]. Notably, such a result was observed both when considering qualitative data [73] and quantitative [74, 75].

The importance of interpersonal relationships for mental health is well stressed by Sullivan's interpersonal theory [76], according to which social interactions are fundamental for positive human functioning and dysfunctional relationships significantly increase psychopathology risk [77]. Moreover, they are the focus of AN cognitive interpersonal maintenance model proposed by Schmidt and Treasure [78], which stresses the role of relationships in pathology onset and maintenance. According to this theoretical framework, anorexic symptoms are maintained intrapersonally by beliefs about the positive function of the illness, and interpersonally by both positive and negative reactions elicited from close others by the physical presentation and behaviors associated with AN (Schmidt & Treasure, 2010,2013. Then, symptoms such as food avoidance might be understood as a strategy to address the need to avoid close relationships that might evoke intense negative emotions. This may be even more the case in Western cultures, where food and eating are inherently social activities: shared meals are often at the heart of social interactions, celebrations, and family gatherings, making eating more than just food and nutrition, but a vital part of social bonding and community.

Following this perspective, Lacan [79] proposed that AN should be understood as a manifestation of a profound struggle with desire and identity, intricately linked to the subject's relationship with the Other (i.e., namely the symbolic representation of societal norms, familial expectations, and interpersonal relationships,[80]). That is, AN might be not merely a struggle with food, but it is a response to Other's demands and desires: food refusal can be then conceptualized as a form of resistance against the symbolic order and the expectations it entails [81]. Importantly, this might also, in some way, reflect their resistance to treatment and lack of trust in care personelle and programs [82].

In light of all these considerations, Cognitive Behavioral Therapy (CBT), the Maudsley Model of Anorexia Nervosa Treatment for Adults (MANTRA; [83, 84]) and Multi-family (MFT-AN,[85]) therapeutic approaches include activities specifically targeted to address social and interpersonal difficulties [111]. Using strategies such as role-playing and behavioral experiments on one hand, and identity exploration and group activities on the other hand, these approaches try to work on the social component of AN. The idea is to allow patients to explore alternative identities, practice and train social skills, and explore alternative behaviors and beliefs. In hospital settings, social activities include psychoeducation groups and peer-discussions. For example, the individuals enrolled in the study by Brusa et al. [27] participated in various rehabilitative activities with intrinsic relational and socializing value (i.e. group discussion, team-work, brainstorming ideas on specific topics, etc.).

The MEDverse proposal [86] represents a conceptual framework that merges VR and augmented reality (AR) technologies within the metaverse to create immersive therapeutic environments tailored for mental health treatment. For example, it is possible to envisage social activities in the metaverse. Such activities might range from role-playing to engaging in conversations about food and body image with virtual characters, behavioral experimentals while practicing eating in virtual social settings, to group therapy sessions with avatars representing other patients and therapists. The technology’s principal advantage lies in its capacity to generate a profound sense of presence and embodiment [112], enabling patients to emotionally and cognitively engage with therapeutic content in a way that transcends traditional interventions.

This heightened sense of immersion manifests through two crucial psychological mechanisms: first, the “place illusion”, where patients genuinely feel present in the virtual environment, and second, the “plausibility illusion”, where they process and respond to virtual events as if they were real [113]. These mechanisms, combined with the capability to induce body ownership illusions through multisensory integration, create a powerful therapeutic platform where patients can safely explore and challenge themselves.

Through carefully designed virtual social scenarios, patients can practice interpersonal interactions in a controlled environment that feels authentically engaging yet remains therapeutically safe. The technology facilitates graduated exposure to challenging social situations, from intimate family meals to broader social gatherings, allowing individuals to develop and refine their social skills while managing anxiety and eating-related behaviors simultaneously. The immersive nature of these experiences, combined with real-time feedback and professional guidance, creates a powerful platform for addressing social isolation, improving communication patterns, and rebuilding damaged relationships—elements that are often central to the maintenance of eating disorders but challenging to address in traditional therapeutic settings [87]. Moreover, the virtual environment can be populated with avatars representing various social roles (family members, peers, colleagues), enabling patients to work through specific interpersonal challenges while developing more adaptive social interaction. The underlying idea is that "exposure to one’s problems and pain as they are experienced in the lives of the others facing you can bring about change of an unforeseen kind with far-reaching consequences" [88, 114].

The findings from this study underscore the critical importance of integrating social relationship work into AN treatment to optimize weight recovery and maintenance outcomes. Even in this context, technologies like IVR offer unique opportunities to address interpersonal challenges through direct experiential learning. The immersive nature of IVR enables patients to engage in social scenarios in real time, bypassing the limitations of traditional therapeutic approaches that rely heavily on mentalization and recall abilities. This is particularly valuable as individuals with AN often struggle with abstract cognitive processing and may benefit more from immediate, embodied experiences.

### Machine learning in clinical practice: pros and cons

In the last few years, ML has emerged as a powerful analysis approach for predicting treatment outcomes and understanding complex patterns in patient data. ML indeed offers several advantages over traditional statistical approaches. First, the latter is primarily used to confirm or refute specific hypotheses and may be more susceptible to overfitting issues when dealing with complex and multidimensional datasets [89], while supervised ML focuses on prediction and provide robust internal validation of model performance through techniques such as k-fold cross-validation, which helps mitigate overfitting concerns and improves generalizability [90]. Second, ML offers greater flexibility in handling diverse data types and model structures.

Supervised ML can be fine-tuned through various parameters and hyperparameters to optimize performance for specific feature types and research questions [91]. Furthermore, ML excels at processing high-dimensional data, allowing it to consider numerous clinical and nonclinical variables simultaneously [53], and it can identify complex, nonlinear relationships among variables without requiring predetermined assumptions [92].

A critical challenge in ML applications for clinical decision-making is the risk of misclassification. Misclassifying patients as likely responders when they are not (false positives) could result in the continuation of ineffective treatments, delaying more suitable interventions. Conversely, incorrectly predicting non-response (false negatives) could lead to the denial of beneficial treatments to patients, impacting their recovery trajectory. These risks underscore the need for rigorous model evaluation and can be mitigated through careful model selection, robust validation techniques, and post hoc explanation methods [93]. It is imperative to acknowledge that ML models should be regarded as decision support systems, wherein the ultimate decision rests with healthcare professionals to ensure patient-centered care.

In conclusion, the adaptability and flexibility of ML allow researchers to tailor their analyses to the unique characteristics of patient data, potentially uncovering new predictors, and to be able to use multidimensional data considering the multifaceted nature of the disorder. This is particularly useful in the AN context, where, as previously seen, multiple factors, physiological markers to psychological traits, and environmental influences can contribute to treatment outcomes. Finally, this data-driven approach has the potential to reveal unexpected connections and patterns in AN data, potentially leading to new insights into the etiology and treatment. In this regard, future studies could also consider non-clinical parameters (e.g., questionnaires on social aspects) and anamnestic factors (e.g., family history of mental health difficulties) to better understand their impact and possible links between them.

However, supervised ML also presents some important limitations. Many of them are like traditional statistics. Both methods can be affected by data quality issues, such as selection bias or measurement error, which can lead to misleading results or poor generalizability. This means that data might be not generalizable across different populations, such as individuals who seek help and those who do not [16]. Additionally, while ML often requires large datasets for optimal performance [94], traditional statistics can also benefit from larger sample sizes to increase statistical power and precision [95]. Lastly, a specific challenge for many ML models in some application areas, such as healthcare, is their “black box” nature [47]. Unlike traditional statistical methods, which often provide interpretable coefficients or odds ratios, complex ML algorithms can produce highly accurate predictions without offering clear explanations for their decision-making processes [96]. This lack of transparency can be problematic in clinical settings, where understanding the rationale behind predictions is crucial for trust and implementation. To address this limitation, the field of explainable AI (XAI) has emerged [97]. XAI aims to make the decision-making processes of ML models more understandable [98]. XAI techniques can provide explanations of model behavior and creation (global level) or individual predictions (individual level, e.g., individual patient), which can be implemented as early as during model creation or used later as posthumous explanations [47]. These insights are particularly important in clinical applications, where interpretability can have a significant impact on the acceptance and effective use of ML models by physicians and patients [99].

## Limitations

This study is situated within the emerging field of research leveraging ML to evaluate treatment effectiveness in mental health (e.g., [100]). In recent years, ML techniques, particularly supervised learning models, have been increasingly used to enhance clinical decision-making and optimize treatment selection in psychiatric disorders, including depression, schizophrenia, and bipolar disorder [115, 116]. However, despite the growing interest in data-driven approaches, it is essential to recognize certain limitations that may impact the interpretation and generalizability of present findings.

First, the limited sample size and imbalance between the two groups being analyzed. However, our sample size aligns both with previous studies in the field [101] and with the minimal number of participants suggested by Figueroa et al. [117] for reliable ML evaluation. Additionally, several methodological strategies were implemented to address such possible limits (e,g., repeated stratified k-fold cross-validation, multicollinearity check, and precision-recall (PR) curve). Future research should validate the model on a larger and more heterogeneous sample to confirm its clinical utility. In this regard, we recommend researchers from different fields collaborate and combine large international datasets to fully exploit ML potential [102]. While we did not preregister this study due to its exploratory nature, adopting open science practices, such as data sharing and preregistration through platforms like the Open Science Framework, can enhance transparency, and reproducibility, as well as the collective effort to advance the field.

Second, we focused on change in BMI as an index of improvement based on Franket et al. [10]. Even though BMI is considered the primary target of AN hospitalization programs, other parameters might be considered as an index of successful treatment (e.g., psychological well-being). Additionally, we conceptualized weight stabilization (ΔBMI = 0) at the same level as an improvement since, in severe conditions and short interventions, the most important aspect is that patients do not lose additional weight and start to stabilize their weight and then improve. This is based on Couturier and Lock [103] who reported that the mean time to AN remission for weight is 11.3 months.

Third, we did not have a follow-up measure as it is not foreseen in the rehabilitation program to have this information. However, the timeframe for assessing treatment success presents a challenge, with some research indicating that the clinical outcome of treatment for AN should be evaluated more than two years post-treatment completion [104]. Although this can be seen as a limitation, it reflects the reality of clinical settings: everyone is unique, just as the expression of their symptoms is, making it impossible to predict the rehabilitation path a person will undertake after discharge. This makes it difficult to maintain a connection with the person for potential follow-ups. Indeed, there are various contexts in which a person might find themselves after discharge, such as communities, hospitals, day hospitals, or returning home. This would make it challenging both to collect and to generalize follow-up data after discharge.

Additionally, in line with the research question, in this study, we focused on psychological and physical features. However, there might be other factors that might influence the treatment outcome, such as the presence of comorbidities, pharmacological treatment, and treatment duration, among others. Future research might consider additional parameters with larger datasets.

Lastly, the present study identifies predictors of treatment efficacy, but it does not explore how these predictors relate to specific therapeutic techniques or treatment outcomes. Thus, we encourage future research to explore ML use to match individuals with the most appropriate interventions based on their unique characteristics.

## Conclusion

The ML potential in psychiatry has generated a great deal of enthusiasm in recent years. This study used ML to predict weight recovery in patients affected by AN attending a multidisciplinary intensive rehabilitation program starting from psychological and physical body parameters. Results revealed that body-self relationship and interpersonal difficulties play a pivotal role in weight restoring, suggesting that therapeutic interventions should focus more on psychological aspects, rather than purely nutritional interventions. If such sensitive topics could be difficult to tackle previously, technology offers new techniques through innovative techniques such as Body Swapping and activities: this would allow to address altered body experience and interpersonal difficulties respectively in immersive and engaging ways. This approach could also take into account the ego-syntonic typical of this disorder, i.e., the patients' difficulty in perceiving reduced food intake as a problem, and its complexity, which is often not centered exclusively on food. By acknowledging these factors, treatment efficacy can be maximized, ensuring that limited resources are strategically used to achieve meaningful clinical outcomes. We argue that this promising area of research could benefit from researchers from different fields working together to create large international datasets that could make a significant contribution to realizing the potential of ML, with the ultimate aim of helping as many people as possible.

## Declarations

## Human ethics and consent to participate

The dataset for the present study was derived from the research by Brusa et al. [27], which was approved by the Ethics Committee of the involved Institution (Reference number: 2022_11_22_05). All participants were volunteers who gave informed written consent before participating in this study,in the case of participants under 18, parents signed the written consent.

## Supplementary Information

Additional file1 (DOCX 302 kb)

## Data Availability

The dataset and the code are available at https://github.com/chiara-pupillo/ML-AN.
