# Supplementary material for: Predicting anorexia nervosa treatment efficacy: an explainable machine learning approach
Source: J Eat Disord. 2025 Jun 2;13:97. doi: 10.1186/s40337-025-01265-3 (PMC12131494; doi:10.1186/s40337-025-01265-3)
Supplement: Supplementary file 1 — Additional file1 (DOCX 302 kb) [file 40337_2025_1265_MOESM1_ESM.docx]

**Supplementary Materials**

**Table 5.** List of abbreviations of predictive variables and their explanation.

|  | Abbreviation | Full Name | Meaning |
| --- | --- | --- | --- |
|  | ***BIA - Bioelectrical impedance analysis*** | | |
| 1 | BIA-ECW | Extracellular water | The ECW index refers to the proportion of water outside the cells. It's calculated as a ratio or percentage of extracellular to the Total Body Water |
| 2 | BIA - BCM | Body Cell Mass | The BCM index is the total mass of all the body cells responsible for consuming oxygen and metabolizing substrates. It includes muscle tissue, organs, and other cellular components actively involved in metabolic processes. |
| 3 | BIA-PA | Phase Angle | The PA index reflects the relationship between resistance (R) and reactance (Xc) in body tissues. It is calculated using the arctangent function of the ratio of reactance to resistance. |
|  | ***EDI3 - Eating Disorder Inventory 3*** | | |
| 4 | EDI3-DT | Drive for thinness | The EDI3_DT measures an excessive concern with dieting, preoccupation with weight, and fear of weight gain. |
| 5 | EDI3-B | Bulimia | The EDI3-B assesses the tendency to engage in binge eating and purging episodes. |
| 6 | EDI3- BD | Body Dissatisfaction | The EDI3-BD evaluates dissatisfaction with body shape and size, particularly concerns about specific body areas. |
| 7 | EDI3-EDRC | Eating Disorder Risk Composite | The EDI3-EDRC is calculated as the sum of DT, B, and BD subscale scores. |
| 8 | EDI3-LSE | Low Self-Esteem | The EDI3-LSE assesses feelings of inadequacy and a lack of self-worth. |
| 9 | EDI3-PA | Personal Alienation | The EDI3-PA measures feelings of isolation, emptiness, and lack of fulfillment in life. |
| 10 | EDI3-II | Interpersonal Insecurity | The EDI3-II evaluates discomfort in social situations and fear of rejection or disapproval from others. |
| 11 | EDI3-IA | Interpersonal Alienation | The EDI3-IA assesses feelings of detachment and lack of connection with others. |
| 12 | EDI3-ID | Interoceptive Deficits | The EDI3-ID measures difficulties in recognizing and accurately identifying internal emotional states and bodily sensations. |
| 13 | EDI3-ED | Emotional Dysregulation | The EDI3-ED evaluates problems with managing and regulating emotions. |
| 14 | EDI3-P | Perfectionism | The EDI3-P assesses the extent to which an individual has high personal standards and a need for flawless performance. |
| 15 | EDI3-A | Asceticism | The EDI3-A measures a tendency towards self-denial, excessive self-discipline, and a belief that self-sacrifice is virtuous. |
| 16 | EDI3-MF | Maturity Fear | The EDI3-MF assesses fear of adult responsibilities and a preference for a more childlike role. |
| 17 | EDI3-IC | Inadequacy Composite | The EDI3-IC is calculated as the sum of LSE and PA subscale scores. |
| 18 | EDI3-IPC | Interpersonal Problems Composite | The EDI3-IPC is calculated as the sum of II and IA subscale scores. |
| 19 | EDI3-APC | Affective Problem Composite | The EDI3-APC is calculated as the sum of ID and ED subscale scores. |
| 20 | EDI3-OC | Obsession Composite | The EDI3-OC is calculated as the sum of A and P. |
| 21 | EDI3-GPMC | General Psychological Disadaptation Composite | The EDI3-GPMC is calculated as the sum of LSE, PA. II, IA, ID, ED, P, and A. |
| 22 | EDI3-IN | Response Incoherence | The EDI3-IN assesses the consistency of responses across the inventory. |
| 23 | EDI3-IF | Infrequency | The EDI3-IF assesses the frequency of atypical or rare responses, helping to identify unusual or inconsistent patterns in how individuals respond to the questionnaire items. |
| 24 | EDI3-NI | Negative Impression | The EDI3-NI assesses respondents’ tendency to present themselves in an overly negative or pathological manner. |
|  | ***SCL90 - Symptom Check List-90*** | | |
| 25 | SCL-90-Somatization |  | tHE SCL-90-Somatization subscale assesses distress arising from perceptions of bodily dysfunction. It includes items related to cardiovascular, gastrointestinal, respiratory, and other systems. |
| 26 | SCL-90-Obsession/Compulsivity |  | The SCL-90-Obsession/Compulsivity subscale measures thoughts, impulses, and actions experienced as unremitting and irresistible but are unwanted. |
| 27 | SCL-90-Interpersonal Sensitivity |  | The SCL-90-Interpersonal Sensitivity subscale reflects feelings of personal inadequacy and inferiority compared to others, including discomfort in interpersonal interactions. |
| 28 | SCL-90-Depression |  | The SCL-90- Depression subscale covers a range of depressive symptoms, including mood and affect, as well as cognitive and somatic aspects. |
| 29 | SCL-90-Anxiety |  | The SCL-90- Anxiety subscale captures symptoms associated with general anxiety, such as nervousness, tension, and panic attacks. |
| 30 | SCL-90-Hostility |  | The SCL-90-Hostility subscale measures thoughts, feelings, and actions characteristic of anger and aggression, including irritability and rage. |
| 31 | SCL-90-Phobic Anxiety |  | The SCL-90-Phobic Anxiety subscale reflects persistent fears of specific situations or objects, as well as agoraphobia and other situational fears. |
| 32 | SCL-90-Psychoticism |  | The SCL-90-Psychoticism subscale measures a broad spectrum of psychotic symptoms, from mild interpersonal alienation to more severe symptoms like hallucinations and thought disorder. |
| 33 | SCL-90-Paranoid Ideation |  | The SCL-90-Paranoid Ideation subscale assesses paranoid thoughts, including suspiciousness, hostility, and fear of loss of autonomy. |
| 34 | SCL-90-SCL Global Index |  | The SCL-90-SCL Global Index subscale provides an overall measure of psychological distress. It is calculated by averaging the scores of all 90 items. |
|  | ***BUT - Body Uneasiness Test*** | | |
| 35 | BUT-GSI (scale A) | Global Severity Index | The BUT-GSI (scale A) is the sum of WP, BIC, A, CSM, and D subscale scores |
| 36 | BUT-WP (scale A) | Weight Phobia | The BUT-WP (scale A) measures the fear of being or becoming fat/gaining weight. |
| 37 | BUT-BIC (scale A) | Body Image Concerns | The BUT-BIC (scale A) assesses worries related to physical appearance. |
| 38 | BUT-A (scale A) | Avoidance Behavior | The BUT-A (scale A) assesses body image-related avoidance behavior. |
| 39 | BUT-CSM (scale A) | Compulsive Self-Monitoring | The BUT-CSM (scale A) assesses compulsive checking of physical appearance. |
| 40 | BUT-D (scale A) | Depersonalization | The BUT-D (scale A) measures the detachment and estrangement feelings toward the body. |
| 41 | BUT-PST (scale B) | Positive Symptom Total | The BUT-PST (scale B) represents the number of body areas that create distress (i.e., body areas with a score greater than 0) |
| 42 | BUT-PSDI (scale B) | Positive Symptom Distress Index | The BUT-PSDI (scale B) is calculated as the sum of scores assigned to each body area. |
|  | ***PGWBI - Psychological General Well-Being Index questionnaire*** | | |
| 43 | PGWBI-Anxiety |  | The PGWBI-Anxiety subscale measures feelings of tension, worry, and nervousness. |
| 44 | PGWBI-Depression |  | The PGWBI-Depression subscale measures feelings of sadness, hopelessness, and lack of interest in daily activities. |
| 45 | PGWBI-Positivity and Wellbeing |  | The PGWBI-Positivity and Wellbeing subscale evaluates positive emotions, satisfaction with life, and overall happiness. |
| 46 | PGWBI-Self-Control |  | The -PGWBI-Self-Control subscale reflects perceptions of one's ability to cope with stress and control one's emotions. |
| 47 | PGWBI-General Health |  | The PGWBI-General Health subsale assesses perceptions of overall physical health and well-being. |
| 48 | PGWBI-Total Score |  | The PGWBI-Total Score subscale is calculated as the sum of the ratings assigned to each item within each dimension. |
|  | ***FMPS - Frost Multidimensional Perfectionism Scale*** | | |
| 49 | FMPS-Concerns Over Mistakes |  | The FMPS-Concerns Over Mistakes subscale measures the extent to which individuals are preoccupied with making mistakes, as well as the fear of criticism and negative evaluations. |
| 50 | FMPS-Organization |  | The FMPS-Organization subscale assesses uncertainty about the correctness of one's actions and the tendency to seek reassurance from others. |
| 51 | FMPS-Personal Standard |  | The FMPS-Personal Standard subscale reflects the setting of high personal standards and the motivation to achieve them. |
| 52 | FMPS-ParentaL Exp. and Parental Criticism | Parental Expectation and Criticism | The FMPS-ParentaL Exp. and Parental Criticism subscale measures perceptions of high parental expectations and criticism and perceived levels of parental criticism and disapproval, particularly related to performance and achievement. |

**Table 6.** Sample descriptives in variables of interest. The table presents descriptives of predictor variables at admission.

| Descriptives questionnaires | | | | | | | |
| --- | --- | --- | --- | --- | --- | --- | --- |
|  | | **Mean (SD)** | | **Minimum** | | **Maximum** | |
| BIA-ECW |  | 15.50 (8.34) |  | 8.60 |  | 57.00 |  |
| BIA-BCM |  | 14.6 (4.73) |  | 1.60 |  | 32.10 |  |
| BIA-PA |  | 4.34 (1.17) |  | 1.30 |  | 7.80 |  |
| BIA-ECWr |  | 14.46 (3.88) |  | 10.40 |  | 26.90 |  |
| BIA-BCMr |  | 15.38 (5.77) |  | 1.90 |  | 38.90 |  |
| BIA-PAr |  | 4.35 (1.07) |  | 1.50 |  | 6.30 |  |
| EDI3-DT |  | 22.29 (7.45) |  | 0 |  | 28 |  |
| EDI3-B |  | 5.62 (7.83) |  | 0 |  | 32 |  |
| EDI3-BD |  | 28.15 (7.24) |  | 15 |  | 40 |  |
| EDI3-EDRC |  | 56.06 (16.48) |  | 20 |  | 96 |  |
| EDI3-LSE |  | 15.93 (5.84) |  | 1 |  | 24 |  |
| EDI3-PA |  | 15.23 (6.56) |  | 2 |  | 28 |  |
| EDI3-II |  | 13.52 (6.82) |  | 0 |  | 26 |  |
| EDI3-IA |  | 12.84 (5.83) |  | 1 |  | 28 |  |
| EDI3-ID |  | 21.27 (8.50) |  | 2 |  | 36 |  |
| EDI3-ED |  | 10.88 (7.20) |  | 0 |  | 37 |  |
| EDI3-P |  | 11.76 (5.10) |  | 0 |  | 24 |  |
| EDI3-A |  | 14.36 (5.78) |  | 4 |  | 28 |  |
| EDI3-MF |  | 17.30 (7.34) |  | 0 |  | 32 |  |
| EDI3-IC |  | 31.16 (11.84) |  | 5 |  | 52 |  |
| EDI3-IPC |  | 26.37 (11.65) |  | 3 |  | 51 |  |
| EDI3-APC |  | 32.02 (13.69) |  | 2 |  | 63 |  |
| EDI3-OC |  | 26.12 (9.29) |  | 5 |  | 51 |  |
| EDI3-GPMC |  | 133.00 (40.04) |  | 46 |  | 237 |  |
| EDI3-IN |  | 12.37 (5.85) |  | 0 |  | 27 |  |
| EDI3-IF |  | 1.77 (1.97) |  | 0 |  | 10 |  |
| EDI3-NI |  | 26.93 (14.24) |  | 1 |  | 82 |  |
| SCL-90-Somatization |  | 1.70 (0.96) |  | 0 |  | 3.92 |  |
| SCL-90-Obsession-Compulsivity |  | 2.12 (0.86) |  | 0.20 |  | 3.90 |  |
| SCL-90-Interpersonal Sensitivity |  | 2.14 (0.92) |  | 0.22 |  | 3.89 |  |
| SCL-90-Depression |  | 2.44 (0.86) |  | 0 |  | 3.92 |  |
| SCL-90-Anxiety |  | 2.08 (0.91) |  | 0 |  | 3.70 |  |
| SCL-90-Hostility |  | 1.20 (0.91) |  | 0 |  | 3.67 |  |
| SCL-90-Phobic Anxiety |  | 1.09 (0.85) |  | 0 |  | 3.00 |  |
| SCL-90-Psychoticism |  | 1.29 (0.66) |  | 0 |  | 3.00 |  |
| SCL-90-Paranoid Ideation |  | 1.85 (0.83) |  | 0 |  | 3.67 |  |
| SCL-90-SCL Global Index |  | 2.23 (3.53) |  | 0.13 |  | 31.19 |  |
| BUT-GSI |  | 3.05 (1.03) |  | 0.44 |  | 4.79 |  |
| BUT-WP |  | 3.54 (1.09) |  | 0.25 |  | 5.00 |  |
| BUT-BIC |  | 3.44 (1.17) |  | 0.22 |  | 5.00 |  |
| BUT-A |  | 2.32 (1.14) |  | 0 |  | 4.67 |  |
| BUT-CSM |  | 2.84 (1.32) |  | 0 |  | 5.00 |  |
| BUT-D |  | 2.77 (1.42) |  | 0 |  | 6.40 |  |
| BUT-PST |  | 21.25 (9.65) |  | 2 |  | 37 |  |
| BUT-PSDI |  | 3.10 (0.72) |  | 1.46 |  | 5.00 |  |
| PGWBI-Anxiety |  | 9.44 (5.31) |  | 0 |  | 24 |  |
| PGWBI-Depression |  | 6.77 (4.25) |  | 0 |  | 14 |  |
| PGWBI-Positivity and Wellbeing |  | 4.97 (3.31) |  | 0 |  | 15 |  |
| PGWBI-Self-Control |  | 6.55 (3.28) |  | 0 |  | 15 |  |
| PGWBI-General Health |  | 7.88 (3-09) |  | 0 |  | 14 |  |
| PGWBI-Vitality |  | 7.41 (4.36) |  | 0 |  | 19 |  |
| PGWBI-Total Score |  | 43.236 (18-73) |  | 5 |  | 87 |  |
| FMPS-Concern Over Mistakes |  | 50.55 (11-42) |  | 22 |  | 70 |  |
| FMPS-Organization |  | 24.56 (5.38) |  | 7 |  | 30 |  |
| FMPS-Personal Standard |  | 24.65 (6-11) |  | 7 |  | 35 |  |
| FMPS-Parental Exp and Parental Criticism |  | 19.72 (8.29) |  | 8 |  | 40 |  |
|  | | | | | | | |

**Table 7**. Evaluation metrics of the Dummy Classifier. The table shows the results for both binary classes.

| **Dummy classifier** | | |
| --- | --- | --- |
|  | *Class 0 (negative)* | *Class 1 (positive)* |
| Accuracy | 0.73 | |
| AUC-ROC | 0.50 | |
| Precision | 0.00 | 0.73 |
| Recall | 0.00 | 1.00 |
| Specificity | / | 0.00 |
| F1-Score | 0.00 | 0.84 |

| **Linear Discriminant Analysis (LDA)** | | |
| --- | --- | --- |
|  | *Class 0 (negative)* | *Class 1 (positive)* |
| Accuracy | 0.32 | |
| AUC-ROC | / | |
| Precision | 0.09 | 0.55 |
| Recall | 0.17 | 0.38 |
| Specificity | / | / |
| F1-Score | 0.12 | 0.44 |

**Table 8**. Multidisciplinary inpatient intensive rehabilitation treatment structure.

| **Activity** | **Frequency** | **Duration** | **Notes** |
| --- | --- | --- | --- |
| *Individual psychological consultation* | 1/week | 45 minutes | Specialized psychologists provide support for psychological distress, and eating pathology |
| *In-group psychotherapy* | 2/week | 1 hour | Specialized psychologists provide support on a group basis working on the disease, trauma, psychological distress, and eating pathology |
| *Dance Movement Therapy* | 1/week | 1 hour | A specialized psychologist provides an intervention based on *Dance Movement Therapy* to improve body image perception |
| *Body image therapy* | 2/week | 30 minutes | Specialized physiotherapists provide interventions to work on body image distortion |
| *Physiotherapy* | 5/week | 30 minutes | Specialized physiotherapists provide tailored physiotherapy treatments |
| *Low intensity physical activities* | 3/week | 30 minutes | Specialized physiotherapists provide gentle gymnastic sessions |
| *Nutritional counselling - individual* | 1/week | 30/45 minutes | Patients received a personalized dietary therapy with progressive caloric intake adjustments throughout her hospitalization. This approach was aimed at addressing the patient’s restrictive eating patterns and facilitating gradual weight gain or arresting further weight loss. The program included regular individualized dietary monitoring and consultations with a dietitian, as well as the use of nutritional supplements (e.g., Meritene cream and Endodien), which were tailored to meet her specific caloric and nutritional needs. The dietary strategy also involved educational courses on nutrition for patients with eating disorders, to promote a better understanding of the relationship between nutrition, weight gain, and overall well-being. |
| *Nutritional counseling - group* | 1/week | 1 hour | Group intervention provided by specialized dietician relative to healthy eating, general nutrition, and core food groups |
| *Assisted meals* | breakfast, snack, lunch, snack, dinner, snack | 30 minutes to 1 hour | Participants eating all together supervised by a dietician |
